# Should Breastfeeding Be Interrupted after Radiological Imaging Examinations? Evidence and Clinical Applications

**DOI:** 10.3390/children11040453

**Published:** 2024-04-09

**Authors:** Şeyma Karatekin, Ebru Şenol, Nalan Karabayır

**Affiliations:** 1 Department of Child Health and Disease, Faculty of Medicine, Samsun University, Samsun 55080, Türkiye; 2 Social Pediatrics Doctorate Programme, Institute of Child Health Department, Istanbul University, Istanbul 34104, Türkiye; ebrusenol18@ogr.iu.edu.tr; 3 Pediatrics Department, International School of Medicine, Istanbul Medipol University, Istanbul 34810, Türkiye; nkarabayir@medipol.edu.tr

**Keywords:** breastfeeding, breast milk, contrast media, CT scan, X-ray, MRI

## Abstract

*Purpose*: Breastfeeding provides optimal growth and development for infants. Lactating mothers may have challenges maintaining breastfeeding, and one of those challenges is being falsely advised to interrupt breastfeeding following radiologic studies. The aim of this study was to evaluate the knowledge, attitudes and experiences of healthcare professionals regarding breastfeeding after radiological imaging studies on lactating mothers. *Method*: In this cross-sectional study, an online survey consisting of 29 semi-structured questions was delivered to radiology technicians and physicians in radiology and pediatrics via social media. Mixed methods were used to analyze responses descriptively. *Results*: Of the 404 participants, 39% (n = 158) were radiology technicians, 31% (n = 125) were pediatricians, 11% (n = 46) were radiologists, 10% (n = 41) were pediatric residents and 8% (n = 34) were radiology residents. Of all healthcare professionals, 91% reported that breastfeeding does not need to be interrupted after ultrasound, 75% X-ray, 56% mammography, 62% non-contrast CT, 18% contrast-enhanced CT, 93% non-contrast MRI and 23% contrast-enhanced MRI. Interruption of breastfeeding was recommended more frequently after contrast-enhanced imaging studies (*p* < 0.01). After contrast-enhanced CT, 54% of participants recommended pumping and dumping for <24 h and 25% for 24–48 h; after contrast-enhanced MRI, these rates were found to be 57% and 20%, respectively. Of the healthcare professionals, 63% reported that their knowledge about management of breastfeeding after radiological studies was not sufficient. *Conclusions*: Situations requiring the interruption of breastfeeding after radiological studies are rare. However, recommendations in clinical practice vary in our country. Increasing the awareness and knowledge of healthcare professionals will prevent breastfeeding from being negatively affected.

## 1. Introduction

The World Health Organization (WHO) recommends breastfeeding up to two years of age or beyond [1]. Most of the positive effects of breastfeeding on infant and mother health are dose-dependent [2]. It is known that even short periods of interruption in breastfeeding may lead to engorgement, ductal narrowing, mastitis, breast refusal and even early termination of breastfeeding [3,4].

Breastfeeding women may need radiologic studies such as ultrasonography (USG), computed tomography (CT), magnetic resonance imaging (MRI) or mammography. It is not uncommon for breastfeeding women to be falsely advised to interrupt breastfeeding after imaging procedures. The interruption of breastfeeding for up to 48 h can frequently be recommended, especially after contrast-enhanced imaging studies [4].

It is critical that breastfeeding recommendations after radiology studies are evidence based and up to date to prevent any unnecessary interruption of breastfeeding. The current recommendations about the continuation of breastfeeding after radiologic imaging studies are summarized in the Academy of Breastfeeding Clinical Protocol #31 [5]. Radiation is used to obtain images in X-ray, mammography and CT studies. Radiation usage in these studies was shown to have no effect on breast milk, and the continuation of breastfeeding was recommended after these studies [5]. In iodinated contrast-enhanced CT, the systemic dose of contrast to the child is less than 0.01% of the maternal dose [5]. In gadolinium-based contrast-enhanced MRI, the systemic dose to the child is less than 0.0004% of the maternal dose [5]. Therefore, there is no need for breastfeeding interruption after contrast-enhanced CT or MRI studies [5,6,7]. In addition, it has been stated that breastfeeding can be continued even with some nuclear medicine imaging methods, such as Technetium-99m mertiatide (Tc-99m MAG3) or Technetium-99m succimer (Tc-99m DMSA) [5].

The sudden cessation of breastfeeding may result in an increased risk of engorgement, mastitis and cow’s milk allergy development especially in infants younger than 6 months. Moreover, the usage of bottles even for a short period of 24 h may cause breastfeeding problems, with the risk of breastfeeding discontinuation. Breastfeeding means more than just feeding. Apart from the optimal growth of the baby, it helps the baby to calm down, regulate body temperature and go to sleep, and it strengthens the mother–baby bond. It is not too hard to anticipate the challenging situation of a mother who had to interrupt breastfeeding suddenly with a baby refusing to feed from a bottle or a cup [3].

There may be delays in the diagnosis of important diseases such as breast cancer in breastfeeding women due to many different reasons [8]. Health professionals’ awareness of the applicability of almost all radiological studies in breastfeeding women, mostly without interrupting breastfeeding, can prevent them from creating a barrier to diagnosis.

Considering the benefits of breastfeeding on infant and maternal health, preventing the unnecessary interruption of breastfeeding after radiologic studies is of major importance. This study aimed to evaluate the knowledge, experience and attitudes of radiology technicians and physicians in radiology and pediatrics regarding breastfeeding continuation in women after radiologic imaging studies.

## 2. Materials and Methods

### 2.1. Design

This cross-sectional descriptive study was conducted with a semi-structured questionnaire which was delivered via social media in May–July 2023. This allowed us to reach more participants from all around the country, thus increasing representation. Ethical approval was obtained from Samsun University Clinical Research Ethics Board: code no. 2023/8/8; date, 26 April 2023.

### 2.2. Setting

Breastfeeding is very common in our country. The Türkiye Demographic and Health Survey 2018 reported that 98% of infants in the country are breastfed, with a median length of 16.7 months. The ratio of infants breastfed until 2 years of age was 34% in the same report [9].

In the country, almost 98% of the births take place in baby-friendly hospitals. A total of 1309 hospitals, including second- and third-level public hospitals, university hospitals and private hospitals, have the baby-friendly title [10]. This study was able to include healthcare professionals from all of these different institutions with baby-friendly titles.

In Türkiye, which has 81 cities, the most crowded city is Istanbul, with 18.6% of the population living in it [11]. This study reached out to participants from 45 different cities, including Istanbul.

### 2.3. Sample

Pediatricians, radiologists, pediatric residents, radiology residents and radiology technicians actively working in radiology units were included in the study. Healthcare professionals were recruited from WhatsApp and Telegram social media pages dedicated to pediatricians, radiologists and radiology technicians. A total of 404 participants responded to the questionnaire in the pre-determined time frame of 3 months.

### 2.4. Data Collection

The semi-structured questionnaire formed by researchers included 29 questions in 3 sections about demographics, knowledge about radiologic imaging studies in breastfeeding women and experience and attitudes about breastfeeding continuation after radiologic studies. While creating questions about breastfeeding and radiological imaging, the Academy of Breastfeeding Medicine protocol 31 was used as a source [5]. The survey was reviewed by the researchers, one of whom is an IBCLC. Questions were entered into an online survey using Google Forms. Before starting the survey, a short section of text and a consent question were included to provide information about the study and obtain informed consent from participants.

#### 2.4.1. Demographic Characteristics of the Participants

The first section contained 10 questions asking participants’ age; gender; occupation, education; years of experience; institution; city of residence; and personal characteristics such as parenthood and longest time period of breastfeeding for their children, if applicable.

#### 2.4.2. Knowledge of Participants about Breastfeeding and Radiologic Imaging Studies

The second section of the questionnaire comprised 7 questions asking participants’ knowledge about the continuation of breastfeeding after radiologic studies and 2 questions about the sources of this knowledge. They were asked about their knowledge about continuing breastfeeding after X-ray imaging, USG, mammography, CT, MRI and contrast-enhanced CT and MRI. The answer options were ‘Yes’, ‘No’ and ‘I don’t know’.

#### 2.4.3. Experience and Attitudes of Participants about Breastfeeding after Radiologic Imaging Studies

The third section contained 10 questions about experience and attitudes of participants. This section started with a question asking how often the participants received questions from breastfeeding women about the continuation of breastfeeding after radiologic studies. Other questions in this section asked about participants’ recommendations about breastfeeding after several imaging studies, including X-ray, mammography, USG, contrast- and non-contrast-enhanced CT, and contrast- and non-contrast-enhanced MRI. To determine the attitudes of participants, questions remarked ‘I recommend continuation of breastfeeding after X-ray studies’. Answer options for this remark included the following: ‘Yes, I agree’; ‘No, I recommend pumping and dumping for <24 h’ or ‘I recommend pumping and dumping for 24–48 h’; and the ‘other’ option gave them the opportunity to write down their individual recommendations. The same question technique was used to learn the recommendations of the participants in other radiologic studies.

### 2.5. Data Analysis

Statistical analysis was conducted using SPSS software version 21 (SPSS Inc., IBM, Armonk, NY, USA). Frequency distributions and percentages were used for categorical variables, while average values along with corresponding standard deviations were reported for continuous numeric variables. The Kolmogorov–Smirnov test was used to test equality of variance and normal distribution in all variables. The Chi-square test was employed to analyze categorical variables. Categorical variables such as breastfeeding recommendations after imaging studies (USG, X-ray, CT and contrast-enhanced CT, and MRI and contrast-enhanced MRI) and occupations were analyzed by the Chi-square test. Statistical significance was accepted at *p* < 0.05.

## 3. Results

Out of 404 participants, 39% (n = 158) were radiology technicians, 31% (n = 125) were pediatricians, 10% (n = 41) were pediatric residents, 11% (n = 46) were radiologists and 8% (n = 34) were radiology residents (Table 1). The mean age of participants was 36.1 ± 8.6 years. The most common city of residency was Istanbul (41%, n = 166). Of the participants, 61.6% had children, and the average longest period of breastfeeding in participants’ children was 17.3 ± 8.6 months (Table 1).

Of all healthcare professionals, 91% (n = 369) reported that breastfeeding does not need to be interrupted after ultrasound (USG), 75% (n = 303) X-ray, 56% (n = 225) mammography, 62% (n = 250) non-contrast CT, 18% (n = 73) contrast-enhanced CT, 93% (n = 375) non-contrast MRI and 23% (n = 94) contrast-enhanced MRI (Table 2).

When participants were asked whether they received questions from mothers about breastfeeding after radiological studies, 54% (n = 25) of radiologists and 49% (n = 77) of radiology technicians answered ‘yes, frequently’. More than half of the total participants (58%, n = 234) encountered such a question within the past year. Of participants, 79% (n = 124) of radiology technicians, 72% (n = 33) of radiologists, 50% (n = 17) of radiology residents, 43% (n = 54) of pediatricians and 15% (n = 6) of pediatric residents reported encountering at least one question about the continuation of breastfeeding after radiologic studies within the past year. 

The experience and attitudes of participants about the continuation of breastfeeding after radiologic studies were different according to occupation (Table 3). The interruption of breastfeeding was recommended more frequently after contrast-enhanced imaging studies (*p* < 0.01). After contrast-enhanced CT, 54% (n = 219) of participants recommend pumping and dumping for <24 h and 25% (n = 99) for 24–48 h. With contrast-enhanced MRI, 57% (n = 231) of participants recommend pumping and dumping for <24 h and 20% (n = 80) for 24–48 h.

No significant differences were detected in participant attitudes according to gender, experience, parental status and continuing breastfeeding after contrast-enhanced and non-contrast CT and MRI (*p* > 0.05). There was a difference in attitudes of participants of different occupations in regard to the continuation of breastfeeding after all radiologic studies except contrast-enhanced MRI (Table 3).

Of all participants, 30% (n = 123) reported receiving training about breastfeeding and radiologic studies and 32% (n = 39) receiving training during their residency period (Table 4). Radiologists had the highest rate (70%, n = 32) of training, and pediatricians had the lowest rate, with 11% (n = 14). Radiology technicians were mostly trained during their undergraduate education, whereas radiologists and pediatricians mostly received training in residency. 

While most of the participants (63%, n = 255) stated that they did not have sufficient knowledge about breastfeeding and radiologic imaging studies, 82% (n = 332) of the them reported that they would want to receive further training on this subject.

## 4. Discussion

Breastfeeding women may need various imaging studies, and the unnecessary interruption of breastfeeding after these studies may negatively affect both the mother and the baby. In our study, recommendation to interrupt breastfeeding was most common after contrast-enhanced imaging, and most of the participants felt that their knowledge on this subject was inadequate.

The Academy of Breastfeeding recommends continuing breastfeeding after X-ray, USG, CT and MRI studies [5]. Although it is known that breastfeeding is safe after X-ray, mammography and US for mastitis, breast abscess or breast masses [5,12,13], 75% of the participants reported that there was no need to interrupt breastfeeding after X-ray and 56% mammography and 92% after USG. However, it is known that breastfeeding is safe after X-rays and mammography [5,12]. 

The American College of Radiology (ACR), American College of Obstetricians and Gynecology and Italian Society of Radiology recommend the continuation of breastfeeding after contrast-enhanced CT and MRI studies [7,14,15]. In a study conducted with radiologists, it was shown that 56% of the participating physicians recommended the interruption of breastfeeding and pumping and dumping of breast milk after contrast-enhanced CT and MRI studies. Since less than 1% of the dose of iodinated contrast material administered to the mother passes into breast milk and less than 1% is absorbed by the baby, it is not expected to cause side effects in the baby [3,4]. Furthermore, the ACR reports that, as iodinated and gadolinium-based contrasts have a plasma half-life of 2 h, they will be totally removed in 24 h in women with normal renal functions [7]. Therefore, women who want to stop breastfeeding after imaging studies should be informed not to interrupt for more than 24 h. In our study, 79% of participants recommended interruption after contrast-enhanced CT and 77% after contrast-enhanced MRI. 

After contrast-enhanced CT, 54% of our participants reported that they recommended pumping and dumping for <24 h, and 25% recommended pumping and dumping for 24–48 h. Similarly, after contrast-enhanced MRI, 57% recommended pumping and dumping for <24 h and 20% recommended pumping and dumping for 24–48 h. The recommendation of interruption of breastfeeding more than 24 h without any scientific evidence in the literature shows that awareness on this subject should be increased among healthcare professionals.

Among the healthcare professionals included in the study, radiology technicians were the ones who most frequently received questions about the continuation of breastfeeding after imaging in the last year. For this reason, it is thought that the subject should be included in the training curriculum for radiology technicians. In addition, post-graduate training should be used to enable radiology technicians to make evidence-based decisions.

It is of great importance that healthcare providers have adequate training on breastfeeding and imaging studies. In our study, more than half (58%) of the healthcare professionals reported receiving questions regarding breastfeeding and radiologic studies within the past year. Colleran et al.’s study reported that 84.5% of the participating radiologists did not receive any training on breastfeeding medicine. Since most of them did not feel sufficiently educated on this subject, it was recommended that breastfeeding medicine should be added to undergraduate and graduate education programs and that easily accessible guides on the subject should be developed [4]. In this study, similarly, 70% of the participants reported the lack of any training on the subject, and 82% were willing to receive a postgraduate training. To support the knowledge and competence of healthcare professionals on breastfeeding and radiological studies, breastfeeding medicine should be included in postgraduate education, in addition to undergraduate education. 

Following the research of Colleran et al., a local guide was prepared for healthcare professionals in Ireland. The necessary steps to support breastfeeding were then determined. This guide states that although breastfeeding is a physiological process, mothers sometimes need support from health professionals. It was emphasized that the need for medication usage or radiological imaging in breastfeeding mothers caused the diagnosis and treatment to be postponed or early termination of breastfeeding. Similarly, while the vaccine administration decisions were made during the pandemic period, the right of breastfeeding women to be vaccinated was postponed [16]. Considering all of this, it is important to prevent delays in breastfeeding women’s diagnosis and treatment processes, while also preventing the unnecessary interruption or termination of breastfeeding.

Strengths of this study: To our knowledge, this is the study on the subject with the largest number of participants, also including pediatricians and radiology technicians in addition to radiologists. The limitation of the study is that it was based on individual personal answers.

## 5. Conclusions

Although the interruption of breastfeeding is not recommended after most radiological imaging studies, there are differences in clinical practice. Providing training to health professionals about breastfeeding and imaging will help prevent early cessation of breastfeeding by increasing awareness and knowledge.

## Figures and Tables

**Table 1:** Demographic characteristics of the participants.

	n = 404	%
Gender		
*Female*	239	59.2
*Male*	165	40.8
Occupation		
*Radiology technician*	158	39.1
*Pediatrician*	125	30.9
*Radiologist*	46	11.4
*Pediatric resident*	41	10.1
*Radiology resident*	34	8.4
Education		
*College*	72	17.8
*Undergraduate*	106	26.2
*Postgraduate*	226	55.9
Professional Experience		
*<5 years*	110	27.2
*5–15 years*	168	41.6
*>15 years*	126	31.2
Institution		
*Own clinic*	11	2.7
*City hospital*	28	6.9
*Private hospital*	42	10.4
*Public hospital*	89	22
*University hospital*	92	22.8
*Training and research hospital*	142	35.1
Parenthood		
*Yes*	249	61.6
*No*	155	38.4

**Table 2:** Knowledge of participants about continuation of breastfeeding after radiologic imaging studies.

Continue Breastfeeding after… (n = 404)	‘Yes’ n (%)	‘No’ n (%)	‘I Don’t Know’ n (%)
X-ray	303 (75)	85 (21)	16 (4)
USG	369 (91)	22 (5)	13 (3)
CT	250 (62)	116 (29)	38 (1)
CT (contrast enhanced)	73 (18)	285 (71)	46 (11)
Mammography	225 (56)	131 (32)	48 (12)
MRI	375 (93)	22 (5)	7 (2)
MRI (contrast enhanced)	94 (23)	254 (63)	56 (14)

USG, ultrasonography; CT, computer tomography; MRI, magnetic resonance imaging.

**Table 3:** Breastfeeding attitudes after imaging methods according to professions.

	Occupation						
Do You Recommend Continuing Breastfeeding after…?	Pediatric Resident	Pediatrician	Radiology Resident	Radiologist	Radiology Technician	Total	*p*-Value
	n (%)	n (%)	n (%)	n (%)	n (%)	n (%)	
USG							
*Yes*	41_a_ (100)	123_a_ (98.4)	34_a,b_ (100)	46_a_ (100)	128_b_ (81)	372 (92.1)	<0.01 *
*No*	0_a_ (0)	2_a_ (1.6)	0_a,b_ (0)	0_a_ (0)	30_b_ (19)	32 (7.9)	
X-ray							
*Yes*	38 ^a^ (92.7)	120_a_ (96)	31_a_ (91.2)	44_a_ (95.7)	62_b_ (39.2)	295 (73)	<0.01 *
*No*	3 ^a^ (7.3)	5_a_ (4)	3_a_ (8.8)	2_a_ (4.3)	92_b_ (58.2)	105 (26)	
*Other ^a^*	0_a_ (0)	0_a_ (0)	0_a_ (0)	0_a_ (0)	4_a_ (2.5)	4(1)	
CT							
*Yes*	33_a_ (80.5)	104_a_ (83.2)	29_a_ (85.3)	42_a_ (91.3)	39_b_ (24.7)	247 (61.1)	<0.01 *
*No*	7_a_ (17.1)	19_a_ (15.2)	4_a_ (11.8)	4_a_ (8.7)	110_b_ (69.6)	144 (35.6)	
*Other ^a^*	1_a_ (2.4)	2_a_ (1.6)	1_a_ (2.9)	0_a_ (0)	9_a_ (5.6)	13 (3.2)	
CT (contrast enhanced)							
*Yes*	10_a_ (24.4)	26_a_ (20.8)	8_a_ (23.5)	14_a_ (30.4)	5_b_ (3.2)	63 (15.6)	<0.01 *
*No*	27_a_ (65.9)	91_a_ (72.8)	24_a_ (70.6)	32_a_ (69.6)	145_b_ (91.8)	319 (79)	
*Other ^a^*	4_a_ (9.8)	8_a_ (6.4)	2_a_ (5.9)	0_a_ (0)	8_a_ (5.1)	22 (5.4)	
MRI							
*Yes*	40_a,b_ (97.6)	119_b_ (95.2)	34_a,b_ (100.0)	43_a,b_ (93.5)	134_a_ (84.8)	370 (91.6)	0.023 *
*No*	1_a_ (2.4)	4_a_ (3.2)	0_a_ (0)	3_a_ (6.5)	19_a_ (12.0)	27 (6.7)	
*Other ^a^*	0_a_ (0)	2_a_ (1.6)	0_a_ (0)	0_a_ (0)	5_a_ (3.2)	7(1.7)	
MRI (contrast enhanced)							
*Yes*	10_a_ (24.4)	29_a_ (23.2)	8_a_ (23.5)	12_a_ (26.1)	20_a_ (12.7)	79 (19.6)	0.102
*No*	28_a_ (68.3)	87_a_ (69.6)	24_a_ (70.6)	34_a_ (73.9)	132_a_ (83.5)	305 (75.5)	
*Other ^a^*	3_a_ (7.3)	9_a_ (7.2)	2_a_ (5.9)	0_a_ (0)	6_a_ (3.8)	20 (5)	
Total	41 (100)	125 (100)	34 (100)	46 (100)	158 (100)	404 (100)	

* *p* < 0.05. ^a^ The other option was ‘I don’t know, I will research the literature, I will look at the contrast agent package insert’. _a,b_ Subscript letters in the tables indicate groups of occupational categories with statistically similar column proportions at the 0.05 significance level.

**Table 4:** Source of education in participants that reported receiving training about breastfeeding and radiologic studies.

	Pediatric Resident	Pediatrician	Radiology Resident	Radiologist	Radiology Technician	Total
	n (%)	n (%)	n (%)	n (%)	n (%)	n (%)
Undergraduate education	0 (0)	0 (0)	0 (0)	1 (3.1)	37 (61.7)	38 (30.9)
Medical school	0 (0)	0 (0)	0 (0)	3 (9.4)	0 (0)	3 (2.4)
Residency	10 (90.9)	5 (35.7)	6 (100)	18 (56.3)	0 (0)	39 (31.7)
Congresses, symposiums	0 (0)	3 (21.4)	0 (0)	3 (9.4)	4 (6.7)	10 (8.1)
Postgraduate education	1 (9.1)	1 (7.1)	0 (0)	1 (3.1)	10 (16.7)	13 (10.6)
Articles or textbooks	0 (0)	5 (35.7)	0 (0)	6 (18.8)	9 (15)	20 (16.3)
Total	11 (100)	14 (100)	6 (100)	32 (100)	60 (100)	123 (100)

## Data Availability

The datasets used and/or analyzed during the current study are available from the corresponding author upon reasonable request. The data are not publicly available as participants were informed that it would be used for scientific purposes only.
